# Inadequate Dietary Nutrient Intake in Patients With Rheumatoid Arthritis in Southwestern Sweden: A Cross-Sectional Study

**DOI:** 10.3389/fnut.2022.915064

**Published:** 2022-06-21

**Authors:** Anna Turesson Wadell, Linnea Bärebring, Erik Hulander, Inger Gjertsson, Helen M. Lindqvist, Anna Winkvist

**Affiliations:** ^1^Department of Internal Medicine and Clinical Nutrition, Institute of Medicine, Sahlgrenska Academy, University of Gothenburg, Gothenburg, Sweden; ^2^Department of Rheumatology and Inflammation Research, Institute of Medicine, Sahlgrenska Academy, University of Gothenburg, Gothenburg, Sweden

**Keywords:** rheumatoid arthritis, energy intake, nutrients, fatty acids, dietary fiber, micronutrients, nutritional requirements

## Abstract

**Background:**

Patients with rheumatoid arthritis (RA), who suffer from impaired physical function and fatigue, may have difficulties with grocery shopping and preparing meals. Also, to improve symptoms, patients often experiment with diets but seldom consult a dietitian. Although this could lead to a nutritiously deprived diet, an up-to-date, thorough description of the nutrient intake in Swedish patients with RA is absent. Here, we investigated the habitual dietary energy and nutrient intake in patients with RA living in southwestern Sweden.

**Materials and Methods:**

Three-day food records performed at two time points during the ADIRA (Anti-inflammatory Diet In Rheumatoid Arthritis) trial, were used. The intake of energy and nutrients was analyzed using The Swedish Food Composition Database.

**Results:**

A total of 62 participants (50 females, 12 males) were included in the study, where 18 participants completed one 3-day food record and 44 participants completed two 3-day food records. Median (IQR) intake of total fat was above or in the upper range of recommendations (females: 37.1 [32.5, 41.7] energy percent (E%), and males: 40.3 [37.5, 42.9] E%). Median (IQR) intake of saturated fatty acids exceeded recommendations (females: 14.9 [12.5, 17.0] E% and males: 15.4 [12.2, 17.0] E%), while median (IQR) carbohydrate and fiber intakes were below recommendations (females: 41.7 [36.3, 45.4] E% and 17.2 [12.8, 20.9] g, respectively, and males: 38.8 [35.2, 40,3] E% and 18.5 [15.7, 21.0] g, respectively). The reported intake of other macronutrients was in line with recommendations. For several micronutrients, e.g., vitamin A and D, folate, and calcium, median intake was below recommended intake. Vitamin A intake was especially low and did not reach lower intake level (LI) for 14 and 17% of females and males, respectively. For females, about 10% did not reach LI for vitamin D, calcium, and riboflavin.

**Conclusion:**

We found that patients with RA residing in southwestern Sweden reported a high intake of saturated fatty acids and low intake of fiber and several micronutrients.

**Clinical Trial Registration:**

[https://clinicaltrials.gov/ct2/show/NCT02941055?term=NCT02941055&draw=2&rank=1], identifier [NCT02941055].

## Introduction

Rheumatoid arthritis (RA) is an autoimmune, chronic disease affecting about 20 million people worldwide and more than 2.3 million in Western Europe (1). The disease primarily affects the joints, where synovitis, cartilage breakdown, and bone erosion cause swelling, stiffness, pain, and disabilities (2). Furthermore, the health-related quality of life (including aspects such as physical function, vitality, and mental health) is reduced in this patient group (3). Because there are few high-quality studies as well as a lack of consistency between previous studies investigating diet and food components in RA, there are currently no specific dietary recommendations for patients with RA. Yet, survey-based research indicates that the patients themselves experience improved or worsened symptoms of certain foods, e.g., fish, berries, and meat (4–7). Although one study investigating dietary intake data from 1997 and 2009 in a Swedish population did not show long-term changes in dietary intake after diagnosis (8), several studies indicate that experimenting with different diets is common in this patient group (5–7). Unfortunately, few patients are offered to consult a dietitian after their RA diagnosis (7).

Impaired physical function, as well as fatigue and a negatively affected mental health, could affect grocery shopping, cooking, and appetite, and thus impact energy and nutrient intake. Several studies have reported energy and nutrient intake in patients with RA, but the results are somewhat disparate (9–24). Some Swedish studies have described a high intake of saturated fatty acids (SFAs) and low intakes of several micronutrients, e.g., vitamin D, vitamin E, and folate, in this patient group (10–12). Outside Sweden, both an inadequate micronutrient intake and a high SFA and low fiber intake have been reported (14–16, 18–24).

There is a lack of up-to-date studies using a high-quality dietary assessment method, i.e., weighed food records, investigating and thoroughly describing nutritional intake in Swedish patients with RA. The ADIRA (Anti-inflammatory Diet in Rheumatoid Arthritis) trial was a randomized crossover trial primarily investigating the effect of a proposed anti-inflammatory diet on disease activity in patients with RA (25). Here, we aimed to investigate the habitual energy and nutrient intake in patients with RA residing in southwestern Sweden, using 3-day weighed food records from the ADIRA trial screening visit and washout period.

## Materials and Methods

### Study Design and Participants

**FIGURE 1:**
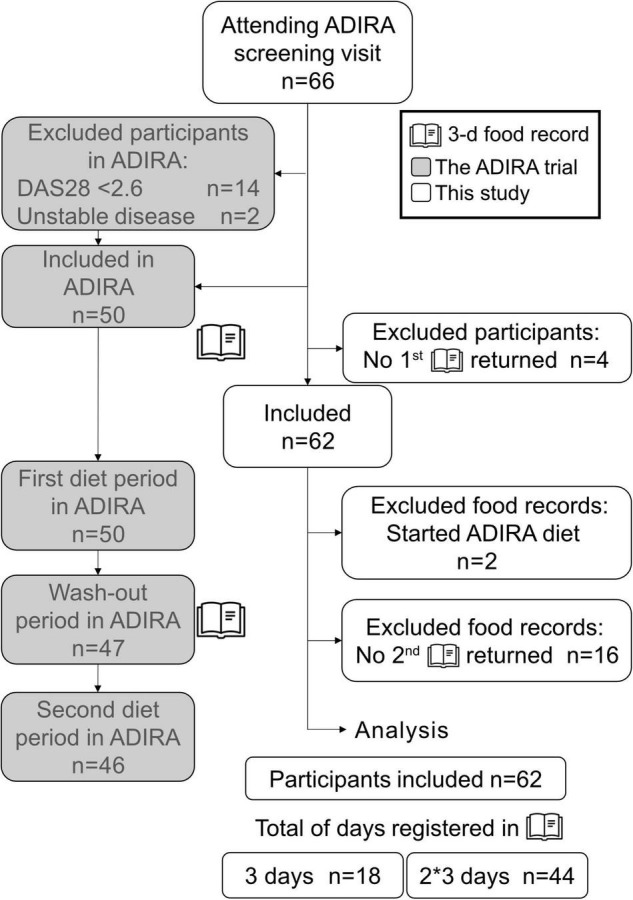
Flowchart showing inclusion of study participants. The gray boxes represent the ADIRA trial, and the white boxes represent this study. ADIRA, anti-inflammatory diet in rheumatoid arthritis; DAS28, disease activity score-28.

In this study, data from the ADIRA trial were used. Details on the study design are described elsewhere (25, 26). In brief, ADIRA was a randomized controlled crossover study in southwestern Sweden, aiming to investigate the effects of a proposed anti-inflammatory diet on RA symptoms. It was performed in two batches, namely, February–December 2017 and August 2017–May 2018. Patients were recruited in two ways, namely, (1) through the Swedish Rheumatology Quality Register (SRQ), where patients residing in the delivery area for the home delivery food chain mat.se, in the Västra Götaland region, were invited to participate in the study, and (2) through posters at the Clinic of Rheumatology, Sahlgrenska University Hospital in Gothenburg, Sweden. The ADIRA inclusion criteria were Disease Activity Score-28 (DAS28) ≥ 2.6 at screening, disease duration >2 years, and clinically stable disease under adequate control (no changes in disease-modifying antirheumatic drugs during the preceding 8 weeks). Exclusion criteria were intolerances to study food or not willing to consume an omnivore diet, other serious illnesses, pregnancy, lactation, and inability to understand the information given. The ADIRA trial was a crossover study with 2 * 10 weeks diet interventions separated by 4 months washout. In this study, all participants attending the screening visit were eligible for inclusion, although they may not have been included in the ADIRA trial. Participants who did not return any food records were excluded from this study and food records performed when the participant had already started any diet in the ADIRA trial were also excluded. For details on study inclusion and exclusion, refer to Figure 1.

### Dietary Assessment

Shortly after the screening visit, participants completed a 3-day food record. For participants later included in the ADIRA trial, this was scheduled to be done before starting the first diet period, and also at the end of the washout period. A dietitian carefully went through the procedure of performing a food record with each participant. Details on the instructions to the participants have been described previously (25). In brief, they were instructed to eat and drink as usual, register their entire food and beverage intake (except for tap water and spices) on three consecutive days (including 1 weekend day), and preferably weigh all items. Participants later not included in the ADIRA trial were asked to send their completed food record by mail. To sort out any uncertainties, a dietitian went through the food record together with the participant over the phone. By using online pictures of different portion sizes [pictures from the booklet Portionsguide (27)], amount of food not weighed or measured by household measuring cups could still be estimated. Participants included in the ADIRA trial were asked to bring their first completed food record to their first visit, and the food record performed during the washout period to their third visit. At both visits, a dietitian went through the food records together with the participant and used portion size pictures if needed (27).

All food records were analyzed by the same dietitian using Dietist Net Pro version 18.12.16 (Kost och Näringsdata AB) and primarily The Swedish Food Composition Database 2017-12-15 (28). When certain food items were missing and not exchangeable, the National Food Composition Database in Finland was used (Fineli 2018-02-28). On three occasions, when a nutritional supplement drink, weight loss powder, and a very small amount of gluten-free flour mix had been consumed, the producers’ own information on nutrient content was used. For the flour mix, information on some micronutrients was, however, missing. Information on food supplement use was collected during a telephone interview before inviting the participants to the screening visit. These were not included in the food record analyses.

If the participant had only used household measuring spoons and cups, and the food database did not provide information about weight from these kinds of measures, the following alternatives, in this order, were used instead, namely, (1) the tables in the key to Portionsguide (29), (2) the dietitian’s own weight measurements, (3) data from Fineli 2018-02-12, (4) Internet sites providing this information, and (5) “normal portion” according to the database used. When it was obvious that a participant used an incorrect number or unit, these were corrected to accurate numbers (e.g., 0.5 dl honey added to 2 dl tea was corrected to 0.5 tbsp).

### Nutrients and Recommendations

When choosing which nutrients to include in this study, two requirements were observed, i.e., (1) a recommended intake (RI) for the nutrient in Sweden and (2) reliable data in The Swedish Food Composition Database 2017-12-15 (28), as well as in the food records. For example, since the participants were not obligated to report salt intake, sodium and iodine intake from the analyses was not reliable and thus not included in this study.

The average daily intake of energy, macronutrients, and micronutrients was investigated and presented as megajoule (MJ), kilocalories (kcal), g, mg, μg, and/or energy percent (E%). In addition, the fiber intake was also reported as g/MJ. Vitamins A and E and niacin intake were reported as retinol equivalents (RE), α-tocopherol equivalents (α-TE), and niacin equivalents (NE), respectively. In The Swedish Food Composition Database, RE are calculated as μ*gretinol*+μ*g*β−*carotene*/12+*othercarotenoids*/24, 1 μg vitamin E = 1 μg α-tocopherol, and NE are the sum of niacin in the food and the niacin formed by the body from the conversion of the amino acid tryptophan (60 mg tryptophan = 1 NE) (30). N-3 fatty acids were calculated as the sum of alpha linolenic acid (ALA), eicosapentaenoic acid (EPA), docosahexaenoic acid (DHA), and docosapentaenoic acid (DPA).

Nordic nutrition recommendations 2012 (31) were used to compare the reported average daily intake to RI, average requirement (AR), and lower intake level (LI). RI “[…] refers to the amount of a nutrient that meets the known requirement and maintains good nutritional status among practically all healthy individuals in a particular life stage or gender group” (31). The definition of AR is “[…] the lowest long-term intake level of a nutrient that will maintain a defined level of nutritional status in an individual” (31) and “[…] LI is defined as a cut-off intake value below which an intake could lead to clinical deficiency symptoms in most individuals” (31).

### Other Assessments

For body mass index (BMI) calculations, participants were weighed in light clothing and without shoes. Details on height measurement are described elsewhere (25). BMI was calculated as kg/m^2^. Details on how DAS28 (a composite score measuring disease activity in patients with RA, including erythrocyte sedimentation rate, number of tender and swollen joints and the patient’s own estimation of their general health on a visual analog scale [VAS]) was calculated have been described previously (25). During the screening visit, participants filled out a lifestyle questionnaire previously described in detail (25). In brief, the questionnaire contained questions about education level, occupational status, tobacco use, etc. For this study, this was only used to describe the study population.

### Statistics

Histograms were used to explore the distribution of the variables. A few variables did not exhibit normal distribution; still, to allow for comparison with the nutrient intake in the Swedish population in general, values are presented both as mean ± standard deviation (SD) and median plus interquartile range (IQR). For categorical variables, number (%) is presented. As males and females tend to have different eating patterns (e.g., males consume more meat and females more fruit and vegetables, possibly leading to different challenges in reaching recommendations for certain nutrients), results are presented stratified by sex. The average daily nutrient intake based on each 3-day food record was calculated. For participants with two 3-day food records, the mean of the daily nutrient intake for those two records was used. When investigating how many of the participants reached RI, AR, and LI, the sex- and age-specific recommendations were used for each individual. The RI for folate is higher for females in reproductive age and both RI and AR for iron are lower for postmenopausal females. As median age for menopause in Swedish females is 52 years old (32), reproductive females were considered to be <52 years old and postmenopausal females were considered to be ≥52 years old. The cutoffs used for reaching RI ranges, RI, AR, and LI, for each nutrient, are described in Supplementary Tables 1, 2. All statistics were performed using IBM SPSS Statistics version 25 (IBM Corp.).

### Ethics Approval and Consent

This study was conducted according to the Declaration of Helsinki and approved by the regional ethical review board in Gothenburg (registration number 976-16, November 2016, and supplement T519-17, June 2017). Written and informed consent was provided by all participants.

## Results

### Study Participants

In total, 62 participants completed a 3-day food record after the ADIRA trial screening visit and thus were included in this study (Figure 1). Of the 62 participants, 18 (29%) completed one 3-day food record and 44 (71%) completed two 3-day food records. The participants with only one completed food record had either not been included in the ADIRA trial (*n* = 12), started an ADIRA diet before initiating their first food and beverage registration (*n* = 2) or dropped out from the ADIRA trial before the second food record was collected after the ADIRA trial washout period (*n* = 4). The majority of the study participants were females (81%), had a university degree (53%), and did not use nicotine (86%; Table 1). The median age was 63 (IQR 53, 71) years, and almost one-third (29%) of the participants were classified with obesity (mean BMI 27.4 ± 5.2 kg/m^2^; Table 1). As for the disease activity, mean ± SD DAS28 was 3.51 ± 1.10 (Table 1), corresponding to a moderate disease activity, although in the lower range of the scale (33). Some participants usually consumed one or several supplements, not prescribed by a physician, e.g., multivitamins, calcium, magnesium, Blutsaft (iron), or n-3 fatty acids.

**TABLE 1:** Sociodemographic characteristics of the study population, i.e., patients with rheumatoid arthritis residing in southwestern Sweden *^a^*.

	All (*n* = 62)	Females (*n* = 50)	Males (*n* = 12)
Age (years), mean ± SD	61 ± 12	61 ± 12	59 ± 14
Age (years), median [IQR]	63 [53, 71]	63 [54, 71]	61 [49, 72]
**Educational level**			
Primary school	10 (16)	7 (14)	3 (25)
Upper secondary school	19 (31)	13 (26)	6 (50)
University degree or equal	33 (53)	30 (60)	3 (25)
**Occupational status**			
Does not work	26 (42)	23 (46)	3 (25)
≤30 h/week	9 (15)	8 (16)	1 (8)
≥31 h/week	27 (44)	19 (38)	8 (67)
**Parents’ birthplace**			
Europe	57 (92)	46 (92)	11 (92)
Africa	1 (2)	1 (2)	1 (8)
Asia	2 (3)	1 (2)	0 (0)
South America	2 (3)	2 (4)	0 (0)
**Current use of nicotine**			
No nicotine use	53 (86)	44 (88)	9 (75)
Cigarettes	3 (5)	2 (4)	1 (8)
Snuff	4 (7)	2 (4)	2 (17)
Other	2 (3)	2 (4)	0 (0)
**The rheumatic disease**			
Disease duration (years), mean ± SD	20 ± 10	19 ± 10	23 ± 11
Disease duration (years), median [IQR]	19 [10, 28]	18 [10, 27]	22 [13, 30]
ACPA and/or RF positive	45 (73)	35 (70)	10 (83)
DAS28, mean ± SD	3.51 ± 1.10	3.50 ± 1.08	3.55 ± 1.25
DAS28, median [IQR]	3.34 [2.87, 4.40]	3.50 [2.60, 4.40]	3.20 [2.94, 4.51]
**Body mass index (BMI)**			
BMI (kg/m^2^), mean ± SD	27.4 ± 5.2	27.2 ± 5.0	27.9 ± 6.0
BMI (kg/m^2^), median [IQR]	26.5 [23.8, 30.9]	26.6 [23.3, 30.9]	26.2 [24.4, 32.1]
Normal weight (BMI 18.5–24.9)	22 (36)	18 (36)	4 (33)
Overweight (BMI 25.0–29.9)	22 (36)	17 (34)	5 (42)
Obese (BMI ≥ 30)	18 (29)	15 (30)	3 (25)

*Values refer to number (%) except when noted otherwise.*

*^a^Patients attending the screening visit in the randomized controlled crossover study ADIRA (Anti-inflammatory Diet In Rheumatoid Arthritis). ACPA, anti-citrullinated protein antibodies; DAS28, disease activity score-28; IQR, interquartile range; RF, rheumatoid factor; SD, standard deviation.*

### Energy and Nutrient Intake

#### Energy and Macronutrient Intake

**FIGURE 2:**
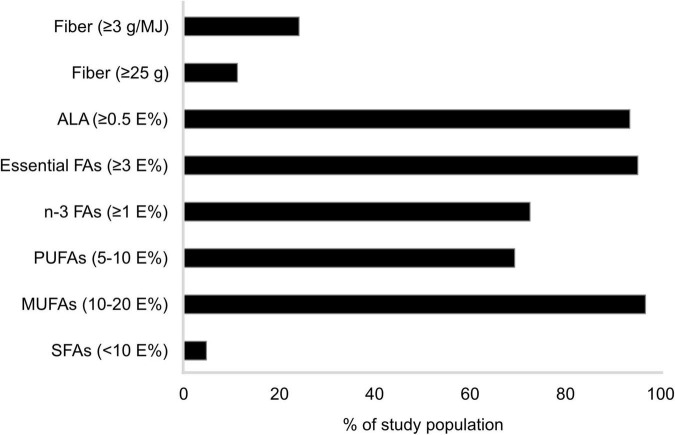
Percentage of study population^1^ (*n* = 62) reporting a fatty acid and fiber intake corresponding to recommendations.^2^ n-3 FA was calculated as the sum of alpha linolenic acid, eicosapentaenoic acid, docosahexaenoic acid, and docosapentaenoic acid. Essential FA was calculated as the sum of alpha linolenic acid and linoleic acid. ^1^Patients with rheumatoid arthritis residing in southwestern Sweden. ^2^Nordic Council of Ministers (31). Nordic Nutrition Recommendations 2012: Integrating nutrition and physical activity [Internet]. Copenhagen: Nordisk Ministerråd; [cited 2021 1 November]. Available from http://urn.kb.se/resolve?urn=urn:nbn:se:norden:org:diva-2561. ALA, alpha linolenic acid; E%, energy percent; FA, fatty acids; MUFA, monounsaturated fatty acids; PUFA, polyunsaturated fatty acids; SFA, saturated fatty acids.

The analysis of the 3-day food records, performed to investigate the participants’ energy and nutrient intake, displayed a median reported energy intake of 7.16 (IQR 5.77, 8.70) MJ for females and 9.02 (IQR 6.96, 10.19) MJ for males (Table 2). The median total fat intake was in the upper range of RI values for females (37.1 [IQR 32.5, 41.7] E%) and males (40.3 [IQR 37.5, 42.9] E%; Table 2) and only 5% had an SFA intake within the recommended range (Figure 2). The reported intake of unsaturated fatty acids was in line with recommendations for the majority of participants (Figure 2). The median intake of carbohydrates was found to be below the RI range (41.7 [IQR 36.3, 45.4] E% and 38.8 [IQR 35.2, 40.3] E% for females and males, respectively; Table 2), with 89% of the study population consuming less than the RI of 25 g fiber/day and 76% not reaching ≥3 g fiber/MJ (Figure 2). Moreover, reported protein and alcohol intakes were in line with recommendations for most participants (Table 2).

**TABLE 2:** Energy and macronutrient intake in patients with rheumatoid arthritis residing in southwestern Sweden *^a^*.

	Females (*n* = 50)		Males (*n* = 12)		
	Mean ± SD	Median [IQR]	Mean ± SD	Median [IQR]	Recommended intake ranges*^b^*
Energy, MJ	7.18 ± 2.00	7.16 [5.77, 8.70]	8.52 ± 1.88	9.02 [6.96, 10.19]	
Energy, kcal	1716 ± 477	1711 [1380, 2077]	2036 ± 451	2154 [1663, 2435]	
Protein, g	67.4 ± 18.5	63.6 [57.5, 74.6]	87.1 ± 33.6	82.0 [64.8, 103.0]	
Protein, E%	16.1 ± 3.3	15.9 [14.0, 17.8]	16.9 ± 3.7	16.0 [14.4, 18.0]	10/15–20*^c^*
Total fat, g	72.1 ± 23.2	72.3 [57.4, 90.7]	90.9 ± 20.6	94.9 [78.5, 106.4]	
Total fat, E%	37.6 ± 6.0	37.1 [32.5, 41.7]	40.5 ± 7.2	40.3 [37.5, 42.9]	25–40
SFA, E%	15.0 ± 3.3	14.9 [12.5, 17.0]	14.3 ± 3.8	15.4 [12.2, 17.0]	<10
MUFA, E%	13.7 ± 2.3	13.9 [12.2, 15.0]	15.2 ± 3.2	15.3 [13.5, 16.5]	10–20
PUFA, E%	5.78 ± 1.52	5.79 [4.59, 7.13]	7.65 ± 2.83	6.61 [5.80, 9.60]	5–10
LA, g	8.17 ± 3.32	8.06 [5.59, 10.39]	12.99 ± 5.28	12.39 [8.77, 17.23]	
LA, E%	4.19 ± 1.15	4.15 [3.20, 5.01]	5.64 ± 2.27	4.81 [4.36, 6.28]	
ALA, g	1.82 ± 0.82	1.7 [1.29, 2.14]	2.63 ± 1.11	2.61 [1.66, 3.22]	
ALA, E%	0.94 ± 0.37	0.89 [0.69, 1.10]	1.12 ± 0.34	1.08 [0.94, 1.38]	≥0.5
Essential FA,*^d^* g	10.0 ± 3.9	9.8 [6.9, 12.4]	15.6 ± 6.2	15.2 [10.1, 21.5]	
Essential FA,*^d^* E%	5.1 ± 1.4	5.0 [3.9, 6.3]	6.8 ± 2.5	6.0 [5.3, 7.8]	≥3
EPA, g	0.17 ± 0.23	0.05 [0.02, 0.28]	0.33 ± 0.31	0.23 [0.11, 0.43]	
DHA, g	0.32 ± 0.37	0.16 [0.06, 0.46]	0.62 ± 0.61	0.38 [0.32, 0.67]	
DPA, g	0.07 ± 0.09	0.03 [0.02, 0.12]	0.15 ± 0.11	0.14 [0.04, 0.23]	
n-3 FA,*^e^* g	2.38 ± 1.16	2.29 [1.52, 2.97]	3.74 ± 2.05	3.51 [2.35, 4.17]	
n-3 FA,*^e^* E%	1.25 ± 0.57	1.15 [0.80, 1.55]	1.57 ± 0.64	1.47 [1.13, 1.73]	≥1
Carbohydrates, g	176 ± 59	168 [129, 229]	192 ± 53	193 [146, 247]	
Carbohydrates, E%	41.2 ± 7.0	41.7 [36.3, 45.4]	37.8 ± 5.8	38.8 [35.2, 40.3]	45–60
Fiber, g	18.0 ± 7.2	17.2 [12.8, 20.9]	20.9 ± 10.7	18.5 [15.7, 21.0]	25–35
Fiber, g/MJ	2.55 ± 0.71	2.53 [1.95, 2.99]	2.52 ± 1.1	2.15 [1.86, 2.89]	≥3
Alcohol, g	8.35 ± 12.15	1.96 [0.00, 12.53]	8.60 ± 8.08	7.33 [0.50, 17.04]	≤10/20*^f^*
Alcohol, E%	3.05 ± 4.21	1.07 [0.00, 5.02]	2.83 ± 2.73	2.38 [0.17, 4.86]	≤5

^a^Energy and macronutrient intake is based on 3-day food records collected at two times during study period; in total, 44 2 * 3-day food records and 18 3-day food records.

*^b^Nordic Council Of Ministers (31). Nordic Nutrition Recommendations 2012: Integrating nutrition and physical activity [Internet]. Copenhagen: Nordisk Ministerråd; [cited 2021 1 November]. Available from http://urn.kb.se/resolve?urn=urn:nbn:se:norden:org:diva-2561.*

*^c^≥65 years old: 15–20 E%.*

*^d^The sum of ALA and LA.*

*^e^The sum of ALA, EPA, DHA, and DPA.*

*^f^≤10 for females, ≤20 for males. ALA, alpha linolenic acid; DHA, docosahexaenoic acid; DPA, docosapentaenoic acid; E%, energy percent; EPA, eicosapentaenoic acid; FA, fatty acids; IQR, interquartile range; LA, linoleic acid; MUFA, monounsaturated fatty acids; PUFA, polyunsaturated fatty acids; SD, standard deviation; SFA, saturated fatty acids.*

#### Micronutrient Intake

**FIGURE 3:**
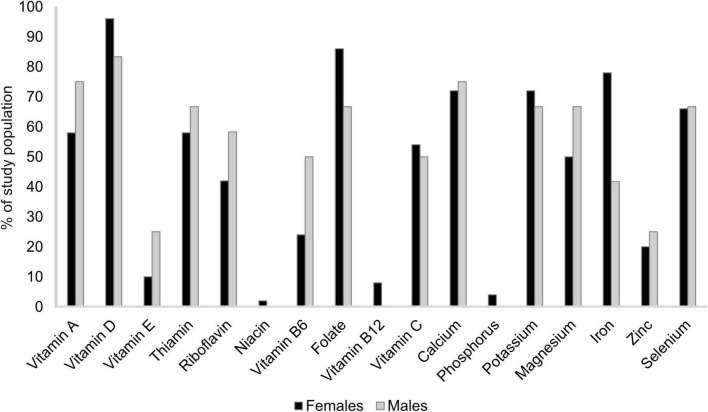
Percentage of study population,^1^ stratified by sex, reporting a micronutrient intake below recommended intake.^2^ ^1^Patients with rheumatoid arthritis residing in southwestern Sweden (females *n* = 50, males *n* = 12). ^2^Nordic Council Of Ministers (31). Nordic Nutrition Recommendations 2012: Integrating nutrition and physical activity [Internet]. Copenhagen: Nordisk Ministerråd; [cited 2021 1 November]. Available from http://urn.kb.se/resolve?urn=urn:nbn:se:norden:org:diva-2561.

For both males and females, median reported intake of several micronutrients was below RI, e.g., vitamins A and D, folate, calcium, and selenium (Table 3). For vitamin D, 96% of females and 83% of males did not reach the RI, folate intake was below RI among 86% of females and 67% of males, 72 vs. 75% did not reach RI for calcium, and about two-thirds of all participants did not reach RI for selenium (Figure 3). Females also consumed iron below RI (median 7.55 [IQR 5.98, 10.23] mg; Table 3) and 78% did not reach RI (Figure 3). Among males, median thiamin, riboflavin, and magnesium intakes were below RI at 1.01 [IQR 0.98, 1.30] mg, 1.30 [IQR 1.10, 1.81] mg, and 323 [IQR 263, 402] mg, respectively (Table 3), and 67% of males did not reach RI for thiamin and magnesium, and 58% for riboflavin (Figure 3).

**TABLE 3:** Micronutrient intake in patients with rheumatoid arthritis residing in southwestern Sweden *^a^*.

	Females (*n* = 50)				Males (*n* = 12)					
	Mean ± SD	Median [IQR]	RI*^b^*	AR*^b^*	LI*^b^*	Mean ± SD	Median [IQR]	RI*^b^*	AR*^b^*	LI*^b^*
Vitamin A, RE	681 ± 254	635 [498, 851]	700	500	400	747 ± 320	643 [521, 916]	900	600	500
Vitamin D, μg	5.2 ± 2.3	5.2 [3.5, 7]	10	7.5	2.5*^c^*	6.9 ± 3	6.4 [4.5, 8.0]	10	7.5	2.5*^c^*
Vitamin E, α-TE	12.1 ± 4.3	10.9 [8.8, 14.7]	8	5	3	15.6 ± 5.9	16.2 [10.2, 18.6]	10	6	4
Thiamin, mg	1.04 ± 0.34	0.95 [0.80, 1.26]	1.1*^d,e^*/1.0*^f^*	0.9	0.5	1.21 ± 0.47	1.01 [0.98, 1.30]	1.4*^d^*/1.3*^e^*/1.2*^f^*	1.2	0.6
Riboflavin, mg	1.29 ± 0.39	1.26 [1.04, 1.47]	1.3*^d^*/1.2*^e,f^*	1.1	0.8	1.45 ± 0.42	1.30 [1.10, 1.81]	1.6*^d^*/1.5*^e^*/1.4*^f^*	1.4	0.8
Niacin, NE	30.8 ± 9.7	29 [24, 34.8]	15*^d^*/14*^e^*/13*^f^*	12	9	39.6 ± 16.1	37.6 [28.2, 47.5]	19*^d^*/18*^e^*/16*^f^*	15	12
Vitamin B6, mg	1.66 ± 0.52	1.57 [1.27, 1.97]	1.2*^d,e^*/1.3*^f^*	1.1	0.8	1.75 ± 0.77	1.5 [1.24, 2.05]	1.5	1.3	1.0
Folate, μg	252 ± 81	236 [192, 294]	400*^d^*/300*^e,f,g^*	200	100	281 ± 126	233 [217, 303]	300	200	100
Vitamin B12, μg	4.71 ± 2.85	4.22 [2.74, 5.42]	2.0	1.4	1	6.14 ± 3.21	4.86 [4.19, 8.73]	2.0	1.4	1
Vitamin C, mg	86.5 ± 50.2	73.8 [48.3, 111.2]	75	50	10	69.2 ± 24.8	74.8 [48.3, 87.7]	75	60	10
Calcium, mg	676 ± 258	668 [465, 857]	800	500	400	660 ± 137	622 [569, 805]	800	500	400
Phosphorus, mg	1,210 ± 351	1,171 [976, 1,456]	600	450	300	1,477 ± 558	1,388 [1,106, 1,559]	600	450	300
Potassium, g	2.86 ± 0.93	2.71 [2.26, 3.26]	3.1		1.6	3.08 ± 0.95	2.96 [2.44, 3.65]	3.5		1.6
Magnesium, mg	287 ± 96	283 [217, 328]	280			358 ± 150	323 [263, 402]	350		
Iron, mg	8.73 ± 4.32	7.55 [5.98, 10.23]	15*^d,e^*/9*^e,f,h^*	10/6*^h^*	5*^h^*	10.51 ± 5.47	9.13 [8.05, 9.78]	9	7	7
Zinc, mg	8.78 ± 3.15	8.02 [7.09, 9.92]	7	5	4	11.68 ± 3.33	11.85 [8.87, 13.12]	9	6	5
Selenium, μg	45.3 ± 19.3	40.1 [31.5, 56.5]	50	30	20	65.1 ± 46	46.5 [39.5, 80.6]	60	35	20

^a^Micronutrient intake is based on 3-day food records collected at two times during study period; in total, 44 2 * 3-day food registrations and 18 3-day food registrations.

*^b^Nordic Council Of Ministers (31). Nordic Nutrition Recommendations 2012: Integrating nutrition and physical activity [Internet]. Copenhagen: Nordisk Ministerråd; [cited 2021 1 November]. Available from http://urn.kb.se/resolve?urn=urn:nbn:se:norden:org:diva-2561.*

*^c^Primarily for > 60 years old.*

*^d^18–30 years old.*

*^e^31–60 years old.*

*^f^61–74 years old.*

*^g^Reproductive age: 400 μg.*

*^h^The lower RI and AR, and LI refer to postmenopausal females.*

*α-TE, alpha-tocopherol equivalents; AR, average requirement; IQR, interquartile range; LI, lower intake level; NE, niacin equivalents; RE, retinol equivalents; RI, recommended intake; SD, standard deviation.*

**FIGURE 4:**
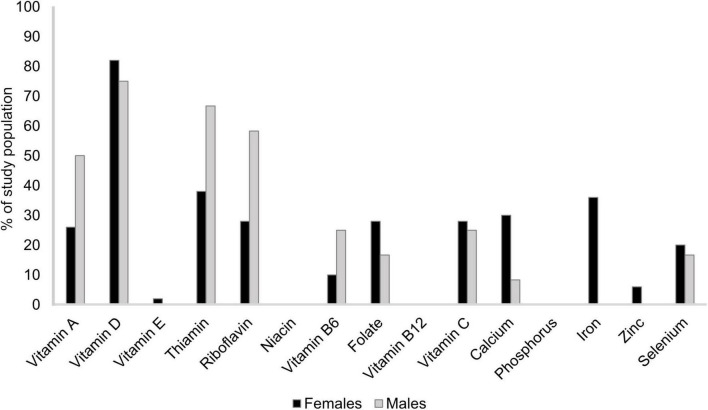
Percentage of study population,^1^ stratified by sex, reporting a micronutrient intake below average requirement.^2^ ^1^Patients with rheumatoid arthritis residing in southwestern Sweden (females *n* = 50, males *n* = 12). ^2^Nordic Council Of Ministers (31). Nordic Nutrition Recommendations 2012: Integrating nutrition and physical activity [Internet]. Copenhagen: Nordisk Ministerråd; [cited 2021 1 November]. Available from http://urn.kb.se/resolve?urn=urn:nbn:se:norden:org:diva-2561.

Less than 75% of the study population reached AR for several micronutrients (Figure 4). Vitamin D intake did not reach AR for 82 and 75% of females and males, respectively. For thiamin, 38% of females and 67% of males did not reach AR. Among males, half of the study population had a reported vitamin A intake not corresponding to AR and 58% did not reach AR for riboflavin. Iron intake among females was below AR for 36% of the study population. Intakes corresponded to LI for most nutrients for the entire study population, with some exceptions. For vitamin A, 14% of females and 17% of males did not reach LI. Among female participants, intakes of vitamin D and calcium (12%), riboflavin (8%), potassium (6%), iron (5% of those ≥52 years old), and thiamin (2%) were also below LI.

## Discussion

In this study, we found that many patients with RA residing in southwestern Sweden reported an inadequate intake of several nutrients. The majority of participants reported a high intake of total fat and almost all participants reported too high an intake of SFA. The intake of carbohydrates was generally low, and fiber intake was lower than recommended for most participants. For many participants, intake of several micronutrients reached neither RI nor AR. Reported intake was especially low for vitamin A, where about 15% of the participants did not even reach LI. For about 10% of the female study population, intake of vitamin D, riboflavin, and calcium were also below LI.

This study is one of very few with the main aim to thoroughly investigate the energy and nutrient intake in Swedish patients with RA. Previous studies reporting nutrient intake in Swedish patients with RA have often focused on other outcomes but have explored dietary intake at baseline, and mainly energy and macronutrient intake (9–11). In our study, we found that the total reported fat intake was high, especially among males, and that the reported carbohydrate and fiber intake was low. This is in contrast to previous Swedish studies showing different macronutrient distributions (9, 10, 12) and higher fiber intake (12). These disparate results could be due to slightly changed recommendations on nutrient intake over time or use of different dietary assessment methods. In addition, Andersson et al. (12) studied an older female population with a lower mean BMI. However, our finding of a high SFA intake is consistent with prior research (10, 12).

As for micronutrient intake, we found that the reported intakes were especially low for vitamin A and, among the female population, also for vitamin D, thiamin, riboflavin, calcium, and potassium. These findings are comparable to those in previous Swedish studies, with some exceptions. Lourdudoss et al. (11) reported approximately the same mean vitamin D intake as in our study and thus below AR. However, folate intake differed between that study and ours, which could be a result from differences in dietary assessment method, and that the study population of Lourdudoss et al. was newly diagnosed with RA. Furthermore, the micronutrients where reported mean intake in our study reached the recommendations were the same as those reported in the study by Andersson et al. except for vitamin A and calcium, which in the study by Andersson et al. were adequate according to recommendations (12). Yet again, these differences could be due to slightly different nutrient recommendations over time and our study population being younger, with a higher BMI.

Studies conducted outside of Sweden mostly display a different macronutrient distribution compared to our study, i.e., more carbohydrates (13–18) and less fat (13–19). Still, a more similar distribution has been reported in a few studies (20, 21). Further, in line with our findings, reported intake of SFA was high (14–16, 19–21) and fiber intake low (14, 15, 21) in several studies. The number of evaluated intakes of micronutrients differs, however, greatly between studies (14, 18, 20, 22–24) as do the results. This could be due to that dietary recommendations as well as food culture often differ between countries. In addition, the dietary assessment methods used varies among the studies. Despite often introducing more bias (34), FFQs were more frequently used than food records. With also our study taken into account, we can, however, conclude that patients with RA often report a too high SFA intake, too low fiber intake, and frequently an inadequate micronutrient intake.

Although patients with RA reported a slightly higher fat intake and lower carbohydrate intake, the reported intake of energy and micronutrients in our study were similar to that of the general Swedish population, according to the national survey Riksmaten 2010-11 (35). Furthermore, both carbohydrate and fiber intakes were lower and SFA intake higher than current recommendations in both Riksmaten and this study. Amcoff et al. concluded that young, Swedish females have a low intake of vitamin D, folate, and iron. Nevertheless, mean intake was below RI for more nutrients in our study, and the difference was especially large for vitamins A, D, and C, and calcium. However, comparisons between our study and the national survey should be made with caution. In contrast to Riksmaten, we did not include patients following a (lacto-ovo) vegetarian diet. Furthermore, Riksmaten was conducted in 2010–2011. Swedish nutrient recommendations and, at least to some extent probably also the food intake patterns, have since changed. This is, however, the best data we currently have on general nutrient intake in the Swedish population.

It is well-established that diet has an impact on health and the risk for developing diseases such as cardiovascular disease (CVD) and cancer. CVD is the leading cause of death in patients with RA and occurs more often in this patient group than in the general population (36). We recently demonstrated that a Mediterranean-like diet rich in unsaturated fatty acids as well as fiber improved blood lipid profiles in patients participating in the ADIRA trial, possibly lowering the risk for atherogenesis (37, 38). Furthermore, calcium and vitamin D play an important role in bone health. Primarily due to glucocorticoid treatment, the risk for osteoporosis is increased in patients with RA (33). Compared to healthy individuals, it has also been shown that this patient group has an altered, less diverse microbiota (39). Even in this aspect, a Mediterranean diet, rich in dietary fiber, seems to be favorable since it has been shown to yield a healthier bacterial composition (40). Hence, dietary intake, and thus nutrient intake, could possibly affect the development of other diseases in patients with RA.

In this study, we reported the percentage of the study population with a reported intake corresponding to the RI, AR, and LI. The RI is determined by taking into account practically all individuals in a population, including those who may have a higher requirement than most individuals (31). Although comparison of mean intake with RI indicates whether a group of individuals eats according to recommendations, it is more accurate to compare the intake with AR and LI to further evaluate the nutrient intake in a certain group of individuals (35). The percentage of patients reaching AR and LI was considerably higher for most nutrients compared to that shown using RI. However, many of our participants were still found to have a very low intake of some micronutrients, especially vitamin A, vitamin D, and calcium. These findings imply that about 10% of our study population have a high risk of developing clinical deficiency symptoms (31). However, many patients with RA are prescribed supplemental folic acid, calcium, and/or vitamin D due to medication side effects (33, 41). Further, some of the participants in our study also consumed non-prescribed nutrient supplements. For this study, we did not include intake of either prescribed nor non-prescribed supplemental vitamins, minerals, or fatty acids, nor did we evaluate serum or plasma nutrient levels. Therefore, it is possible that the total nutrient intake including supplements still covered the individual requirement for some participants.

This study has some limitations we want to address. First, we gave participants thorough instructions on how to perform a food record and emphasized the weighing of food items. Nevertheless, this was not always possible for the participants and on such occasions, errors could possibly have occurred, e.g., incorrect portion size quantification by the participant or incorrect conversion to weight by the dietitian (34). Second, some nutrients (especially vitamin A) often require more than 3 or 6 days of food and beverage registration to capture the true habitual intake in a group (42). The number of days depends on the number of individuals as well as the intra- and/or inter-individual intake variability and is therefore highly dependent on the population studied. However, a longer registration period could have been too much of an effort for the participants and thereby led to missing data. Third, supplement use was common among the participants and therefore total nutrient intake may be higher than the dietary nutrient intake reported here. As we did not have sufficient data of good quality on supplement use, we were unable to perform sensitivity analyses to investigate this further. Finally, underreporting (register less food and beverages than was actually consumed or eating less than usual because of the registration *per se*) may have occurred (43), causing reported intakes of both energy and nutrients to be lower than actual intake (44). However, when analyzing the food records, none of the participants reported an intake less than 500 or 800 kcal; levels that are often used as the lowest acceptable energy intakes for females and males, respectively, in epidemiological studies (44). Furthermore, reported energy intakes were not lower than in other studies on patients with RA or in the general Swedish population.

There are also several strengths. First of all, we used the most precise method to assess nutrient intake, i.e., weighed food records (34). Second, most participants performed the food records at two times and food records were performed during different seasons. Third, when collecting the completed food records, we went through each day carefully with the participant to sort out any uncertainties. Fourth, the same dietitian analyzed all food records, eliminating possible between-examiner errors. Finally, we used SRQ to recruit participants. The gender distribution and age in our study population represent that of the Swedish population with RA (45). In addition, the participants had a disease activity similar to that of the target population 1 year after diagnosis (46). These factors indicate a successful recruitment.

## Conclusion

Patients with RA are a nutritionally vulnerable group, and, in this study, we found that this patient group in southwestern Sweden reported a high intake of SFAs, and low intake of several micronutrients and fiber. Routinely offering a dietitian consultation at the time of diagnosis would likely improve nutritional intake for these patients and possibly lower the risk for comorbidities.

## Data Availability Statement

The raw data supporting the conclusions of this article will be made available by the authors, without undue reservation.

## Ethics Statement

The studies involving human participants were reviewed and approved by the Regional Ethical Review Board in Gothenburg, Sweden. The patients/participants provided their written informed consent to participate in this study.

## Author Contributions

AW, HL, LB, and IG designed the study. ATW, LB, HL, AW, and EH conducted the research. ATW analysed the data, wrote the manuscript, and had primary responsibility for the final content. All authors assisted in interpreting the data and have read and approved the final manuscript.

## Conflict of Interest

The authors declare that the research was conducted in the absence of any commercial or financial relationships that could be construed as a potential conflict of interest.

## Publisher’s Note

All claims expressed in this article are solely those of the authors and do not necessarily represent those of their affiliated organizations, or those of the publisher, the editors and the reviewers. Any product that may be evaluated in this article, or claim that may be made by its manufacturer, is not guaranteed or endorsed by the publisher.

## Abbreviations

ADIRA: anti-inflammatory diet in rheumatoid arthritis
DAS28: disease activity score-28
RA: rheumatoid arthritis.
