# Supplementary material for: Inadequate Dietary Nutrient Intake in Patients With Rheumatoid Arthritis in Southwestern Sweden: A Cross-Sectional Study
Source: Front Nutr. 2022 Jun 21;9:915064. doi: 10.3389/fnut.2022.915064 (PMC9255550; doi:10.3389/fnut.2022.915064)
Supplement: Supplementary file 1 [file Data_Sheet_1.PDF]

Supplementary Material

SUPPLEMENTARY TABLE 1 | Cut-offs used for reaching recommended intake ranges for the macronutrients[1].

	Recommended intake range
Protein, E%	
<65 years old	9.5-<20.5
≥65 years old	14.5-<20.5
Total fat, E%	24.5-<40.5
Saturated fatty acids, E%	<9.5
Monounsaturated fatty acids, E%	9.5-<20.5
Polyunsaturated fatty acids, E%	4.95-<10.5
n-3 fatty acids, E%	≥0.95
Essential fatty acids, E%	≥2.95
Alpha linolenic acid, E%	≥0.495
Carbohydrates, E%	44.5-<60.5
Fiber, g	≥24.95
Fiber, g/MJ	≥2.995
Alcohol, g	
Females	<10.05
Males	<20.05
Alcohol, E%	<5.05

[1] Based on the Nordic Nutrition Recommendations 2012 (Nordic Council Of Ministers. Nordic Nutrition Recommendations 2012: Integrating nutrition and physical activity [Internet]. Copenhagen: Nordisk Ministerråd; 2014 [cited 2021 1 Nov]. Available from: http://urn.kb.se/resolve?urn=urn:nbn:se:norden:org:diva-2561)
E%, Energy percent

SUPPLEMENTARY TABLE 2 | Cut-offs used for reaching RI, AR, and LI for the micronutrients[1]

	RI		AR		LI	
	**Females**	**Males**	**Females**	**Males**	**Females**	**Males**
Vitamin A, RE	≥699.5	≥899.5	≥499.5	≥599.5	≥399.5	≥499.5
Vitamin D, µg	≥9.95	≥9.95	≥7.45	≥7.45	≥2.45	≥2.45
Vitamin E, α-TE	≥7.995	≥9.995	≥4.995	≥5.995	≥2.995	≥3.995
Thiamin, mg	≥1.095[2,3]<br>≥0.995[4]	≥1.395[2]<br>≥1.295[3]<br>≥1.195[4]	≥0.895	≥1.195	≥0.495	≥0.595
Riboflavin, mg	≥1.295[2]<br>≥1.195[3,4]	≥1.595[2]<br>≥1.495[3]<br>≥1.395[4]	≥1.095	≥1.395	≥0.795	≥0.795
Niacin, NE	≥14.95[2]<br>≥13.95[3]<br>≥12.95[4]	≥18.95[2]<br>≥17.95[3]<br>≥15.95[4]	≥11.95	≥14.95	≥8.95	≥11.95
Vitamin B_6, mg	≥1.195[2,3]<br>≥1.295[4]	≥1.495	≥1.095	≥1.295	≥0.795	≥0.995
Folate, µg	≥399.5[2,5]<br>≥299.5[3,4]	≥299.5	≥199.5	≥199.5	≥99.5	≥99.5
Vitamin B_{12}, µg	≥1.995	≥1.995	≥1.395	≥1.395	≥0.995	≥0.995
Vitamin C, mg	≥74.95	≥74.95	≥49.95	≥59.95	≥9.95	≥9.95
Calcium, mg	≥799.5	≥799.5	≥499.5	≥499.5	≥399.5	≥399.5
Phosphorus, mg	≥599.5	≥599.5	≥449.5	≥449.5	≥299.5	≥299.5
Potassium, g	≥3.095	≥3.495			≥1.595	≥1.595
Magnesium, mg	≥279.5	≥349.5				
Iron, mg	≥14.995[2,3,6]<br>≥8.995[3,4,6]	≥8.995	≥9.995<br>≥5.995[6]	≥6.995	≥4.995[6,7]	≥6.995
Zinc, mg	≥6.995	≥8.995	≥4.995	≥5.995	≥3.995	≥4.995
Selenium, µg	≥49.95	≥59.95	≥29.95	≥34.95	≥19.95	≥19.95

[1]*Based on the Nordic Nutrition Recommendations 2012 (Nordic Council Of Ministers. Nordic Nutrition Recommendations 2012: Integrating nutrition and physical activity [Internet]. Copenhagen: Nordisk Ministerråd; 2014 [cited 2021 1 Nov]. Available from: http://urn.kb.se/resolve?urn=urn:nbn:se:norden:org:diva-2561)*

[2] *<31 years old*

[3] *31-<61 years old*

[4] *≥61 years old*

[5] Reproductive age (i.e., <52 years old)
[6] Post-menopausal women (i.e., ≥52 years old)
[7] No LI for women of fertile age (i.e., <52 years old)
α-TE, Alpha-tocopherol equivalents; AR, Average requirement; LI, Lower intake level; NE, Niacin equivalents; RE, Retinol equivalents; RI, Recommended intake
